# Dual assurance for healthcare and future education development: normalized assistance for low-income population in rural areas—evidence from the population identification

**DOI:** 10.3389/fpubh.2024.1384474

**Published:** 2024-11-19

**Authors:** Xiaoyan Peng, Yanzhao Zeng, Yanrui Chen, Huaxing Wang

**Affiliations:** ^1^School of Government, Sun Yat-sen University, Guangzhou, China; ^2^School of Economics and Statistics, Guangzhou University, Guangzhou, China; ^3^School of Public Administration, Guangzhou University, Guangzhou, China; ^4^Institute of Urban Development and Strategy, Law School, Research Center for Digitalization and Rural Development, Hangzhou City University, Hangzhou, China; ^5^School of Economics, Zhejiang University, Hangzhou, China

**Keywords:** rural low-income population, healthcare, future education, target population identification, normalized assistance

## Abstract

**Introduction:**

This study aims to explore the relationship between healthcare and future education among the rural low-income population, using *J* City in Guangdong Province as the focal area. Addressing both healthcare and educational concerns, this research seeks to provide insights that can guide policy and support for this demographic.

**Methods:**

Utilizing big data analysis and deep learning algorithms, a targeted intelligent identification classification model was developed to accurately detect and classify rural low-income individuals. Additionally, a questionnaire survey methodology was employed to separately investigate healthcare and future education dimensions among the identified population.

**Results:**

The proposed model achieved a population identification accuracy of 91.93%, surpassing other baseline neural network algorithms by at least 2.65%. Survey results indicated low satisfaction levels in healthcare areas, including medical resource distribution, medication costs, and access to basic medical facilities, with satisfaction rates below 50%. Regarding future education, issues such as tuition burdens, educational opportunity disparities, and accessibility challenges highlighted the concerns of rural low-income families.

**Discussion:**

The high accuracy of the model demonstrates its potential for precise identification and classification of low-income populations. Insights derived from healthcare and education surveys reveal systemic issues affecting satisfaction and accessibility. This research thus provides a valuable foundation for future studies and policy development targeting rural low-income populations in healthcare and education.

## 1 Introduction

In modern society, healthcare and education development are closely intertwined, collectively shaping the future of society. Individuals in good health are more likely to access high-quality education, and well-educated people generally possess greater awareness and capabilities to maintain their health (1, 2). Therefore, a thorough exploration of the correlation between healthcare and future education is crucial for building a sustainable and thriving society.

Due to regional economic conditions and insufficient medical resources, the rural low-income population faces unique healthcare and education needs (3). Challenges for this group include poverty, unequal access to medical services, and a lack of educational opportunities (4, 5). Addressing the issues of the rural low-income population is crucial for individual survival and development and directly impacts the sustainable progress of the entire society.

Internationally, extensive research has shown that health directly influences individuals’ learning abilities, career development, and social participation. For instance, Qiu et al. (6) reviewed the application, challenges, and future prospects of large artificial intelligence models in health informatics. They emphasized that the widespread application of these models in the healthcare domain directly influenced individuals’ health conditions, thereby significantly impacting learning abilities, career development, and social participation. Shehab et al. (7) highlighted the latest machine learning methods in healthcare applications. The extensive application of these methods in the healthcare domain has enhanced the accuracy of medical diagnoses and profoundly impacted individuals’ health and medical conditions. This influence extends to aspects such as learning, career, and social participation. Van Helden et al. (8), through a gender perspective, explored the impact of occupational shocks on career development. Gender differences and their association with career trajectories directly relate to individual learning and career development, highlighting the crucial role of gender in the professional domain. Jo et al. (9) found that the pressure of adapting to family culture directly influenced individual learning and career choices in the career development of multicultural adolescents from Korean families, revealing the significant impact of cross-cultural backgrounds on career development. Ronaldson et al. (10) discovered associations among multiple chronic conditions, depressive symptoms, and social participation in adults over 50. Health conditions directly influence individuals’ social participation, affecting learning, career, and overall quality of life. Baeriswyl and Oris (11) found that social participation was a key factor in the learning and career development of older adults, highlighting the impact of social inequality on the lives of older people. Prieur Chaintré et al. (12) analyzed the impact of hearing loss on the social participation of older adults, demonstrating a direct correlation between hearing health and social participation in the older adult. A review of the above literature found that valuable lessons can be drawn from advanced countries in the research on the relationship between healthcare and education. By thoroughly investigating the role of population identification mechanisms in assistance, this work provides new theoretical and practical support for enhancing the precision and effectiveness of assistance efforts.

In conclusion, this work selects *J* City in Guangdong Province as the research context to explore the practical application and effectiveness of population identification mechanisms in assisting the rural low-income population. By delving into the advantages of such mechanisms, this work aims to gain a deep understanding of the healthcare and education conditions of the rural low-income population, providing robust support for the formulation of relevant policies.

The research innovation and contribution lie in the exploration of the correlation between healthcare and future education development while introducing the population identification mechanism to offer an innovative and more precise approach to assisting the rural low-income population. Through a comprehensive analysis of this mechanism’s application, the work provides new insights and contributions for future policy formulation and societal practices.

## 2 Method

### 2.1 Analysis of the correlation between healthcare and future education for the rural low-income population

**Figure 1:**
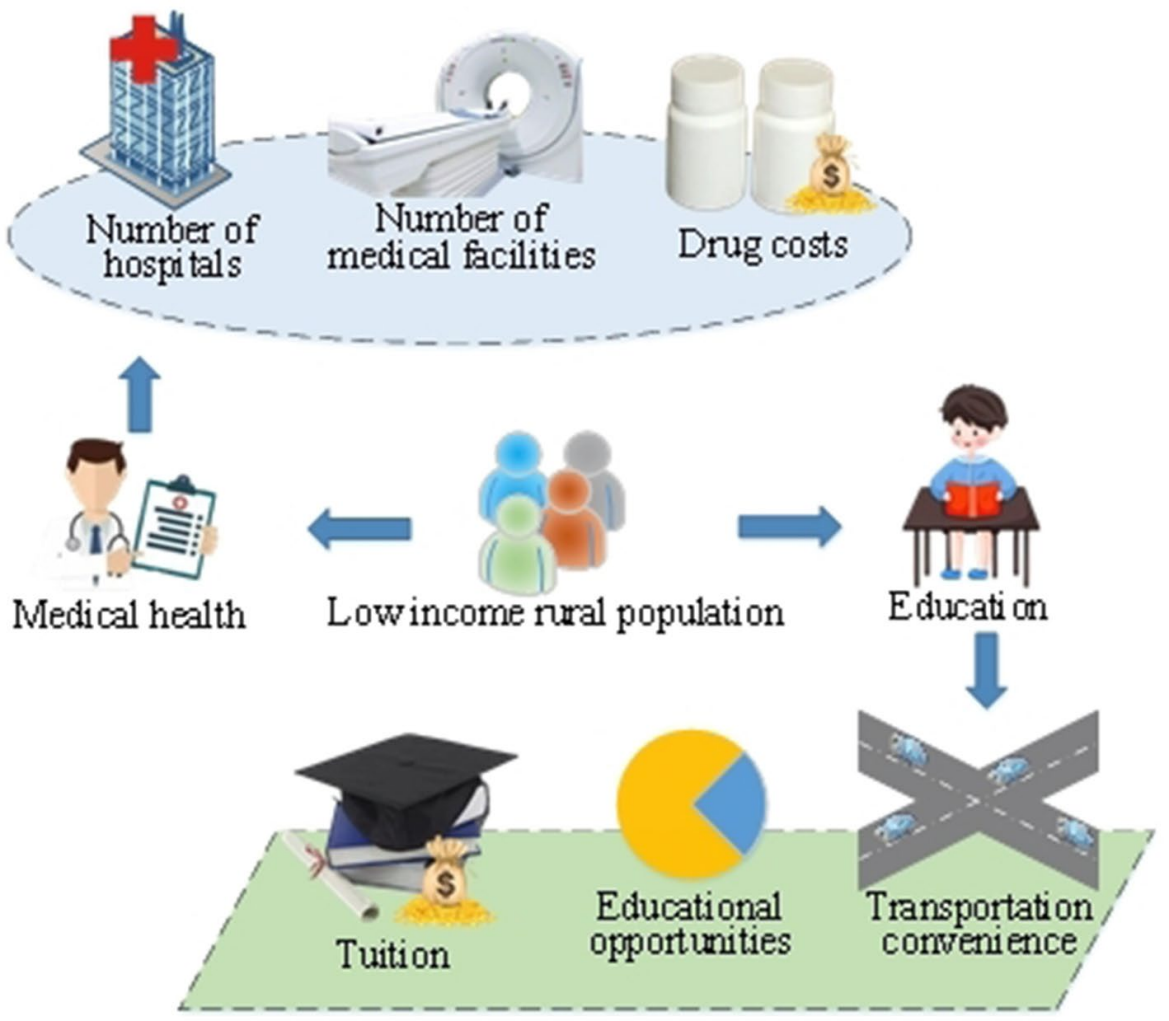
Illustration of factors influencing healthcare and future education for the rural low-income population.

The healthcare and future education of the rural low-income population are two closely intertwined critical domains directly impacting their survival, development, and social participation (13–15). A comprehensive understanding of the specific conditions in these two domains can better grasp their correlation, providing strong support for subsequent research. Figure 1 illustrates the factors to be considered in the healthcare and future education of the rural low-income population.

Figure 1 illustrates the multiple challenges faced by the healthcare of the rural low-income population. Due to constraints in regional economic conditions and inadequate healthcare resources, this demographic often encounters issues such as unequal access to medical services, difficulty in obtaining health information, and a heavy burden of treatment costs. Common health problems may include the prolonged medication needs of chronic patients and a lack of basic medical facilities (16, 17). These factors directly impact the health status of the rural low-income population, potentially influencing their learning abilities, career development, and social participation.

In terms of education, the rural low-income population often faces challenges in future education due to economic difficulties and insufficient educational resources. This includes a lack of educational opportunities, heavy tuition burdens, and inconvenient transportation. As education is closely linked to career development, the educational level of the rural low-income population directly affects their future employment opportunities and career choices (18, 19). Therefore, addressing the healthcare and future education issues of the rural low-income population is crucial for their individual survival and development, and it holds significant importance for the overall sustainable progress of society.

### 2.2 Research design

**Figure 2:**
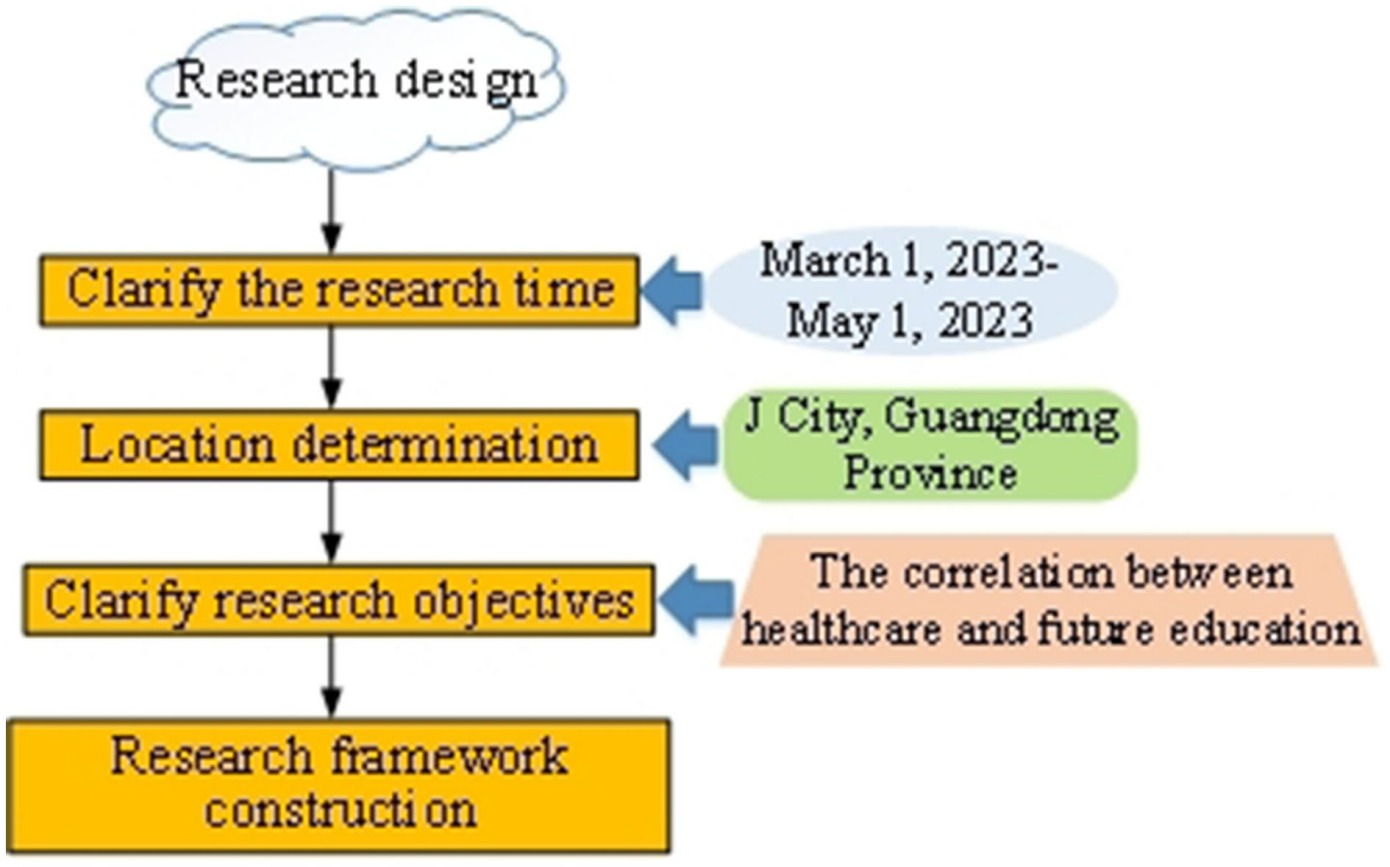
Schematic diagram of the research design stage.

The research design is a crucial step in ensuring the smooth implementation of the research and the comprehensive and systematic achievement of its objectives (20–22). This work focuses primarily on *J* City in Guangdong Province. A multi-stage research design is made to gain in-depth insights into the correlation between healthcare and future education for the rural low-income population. Figure 2 illustrates the stages of the research design.

In Figure 2, the first step involves specifying the research time and location. This work takes *J* City in Guangdong Province as the research area, selecting representative rural areas for investigation from March 1, 2023, to May 1, 2023, ensuring the research results have a certain degree of universality. A more comprehensive understanding of the similarities and differences in the healthcare and future education conditions of the rural low-income population can be obtained by conducting longitudinal and cross-sectional comparisons across different regions.

Next, the research objectives are defined. This work aims to gain in-depth insights into the correlation between healthcare and future education for the rural low-income population. Therefore, core questions that need to be addressed are clearly outlined, including the distribution of medical resources, tuition burdens, and disparities in educational opportunities. This contributes to ensuring the research’s directionality and substance. In this research design, data are primarily collected through a questionnaire survey.

To ensure that the survey methods do not introduce bias in healthcare and future education, several measures are implemented to guarantee the fairness and accuracy of data collection. First, the questionnaire design is based on a comprehensive literature review, ensuring that the questions are thorough and neutral, thus avoiding leading questions and minimizing bias arising from poorly designed questions. Second, a five-point Likert scale is employed, which allows for a detailed capture of respondents’ attitudes and opinions while reducing the likelihood of extreme responses, thereby enhancing the authenticity of the data. Additionally, all participants are involved in the survey on a completely voluntary basis and are explicitly informed of the research objectives and privacy protection measures before filling out the questionnaire, ensuring their informed consent. The anonymity and confidentiality of the data collection process are also maintained, further enhancing the credibility of the results. Through these measures, the impartiality of the survey methods is ensured. This prevents bias in the data collection processes related to healthcare and future education, thereby providing authentic and reliable data support for the research. Table 1 presents the questionnaire items.

**Table 1:** Items of questionnaire survey.

Dimension		Question	Strongly agree	Agree	Neutral	Disagree	Strongly disagree
Healthcare	Distribution of medical resources	A1: I believe the service quality of nearby medical institutions is high					
		A2: I believe that nearby medical institutions have well-equipped facilities					
		A3: Hospitals or clinics around me are very close to my home					
		B1: Treating chronic illnesses does not pose a significant economic burden on me					
		B2: I feel a heavy burden in treating chronic illnesses due to medication costs					
	Distribution of basic medical facilities	C1: There is a community health station or clinic near my home					
		C2: I feel that the services of the community health station or clinic are timely					
		C3: I am satisfied with the service quality of the community health station or clinic					
Future education	Tuition burden	D1: My family’s monthly income is sufficient to cover my children’s tuition					
		D2: The tuition fees for my children at school are relatively low					
		D3: I believe that the burden of my children’s tuition fees imposes significant financial pressure on the family					
	Disparities in educational opportunities	E1: I feel that the schools in my area have teaching staff comparable to urban schools					
		E2: I believe that the schools in my area have relatively good facilities					
		E3: Whether children in the area can access better educational opportunities mainly depends on the family’s economic situation					
	Transportation conditions	F1: The transportation method for my children to go to school is relatively convenient					
		F2: My children’s transportation situation has a relatively minor impact on their academic performance					
		F3: The transportation situation for my children to go to school causes significant trouble for the family					

Finally, the research framework is established. The research framework serves as the blueprint for the entire study, guiding the systematic data collection and analysis. This work uses a questionnaire survey to incorporate factors related to healthcare and future education, and clearly defines the relationships between these factors. The questionnaire covers healthcare-related issues, including the distribution of medical resources, medication costs for chronic patients, and the distribution of basic medical facilities (23). It also addresses core issues in future education, such as tuition burden, disparities in educational opportunities, and transportation conditions (24, 25). The questionnaire design process is primarily divided into three stages: literature review, initial draft design, expert consultation and revision, and final draft.

1. During the literature review, worldwide relevant literature on the healthcare and future education of rural low-income populations is comprehensively reviewed. The data are systematically organized and analyzed, providing a theoretical foundation for questionnaire design.
2. In the initial draft design of the questionnaire, based on the literature review results, a survey questionnaire covering multiple dimensions is constructed, including key issues in healthcare and future education. After completing the initial draft, academic experts in the field are invited to review it. Based on their feedback, relevant modifications are made, resulting in the first draft of the questionnaire.
3. Finally, the first draft of the questionnaire is further revised based on the feedback from experts, ensuring its content and structure are more logical and scientifically grounded.

This survey questionnaire uses the Likert five-point scale method, with response options categorized as strongly agree = 5, agree = 4, neutral = 3, disagree = 2, and strongly disagree = 1. The entire survey process strictly adheres to ethical principles and does not involve personal privacy. Participants are all 18 years old or older, and their participation is conducted with their consent. The questionnaire is not open to the public and is used solely for research purposes.

### 2.3 Ethical approval

This study was approved by the Academic Ethics Review Committee of Hangzhou City University, China, on November 15, 2023. Our study did not involve animal or human clinical trials and was not unethical. In accordance with the ethical principles outlined in the Declaration of Helsinki, all participants provided informed consent before participating in the study. The anonymity and confidentiality of the participant guaranteed, and participation was completely voluntary. Participants voluntarily click on the link to fill out the questionnaire. Before filling out the questionnaire, they have been informed of the research purpose and informed that “submitting answers” is considered informed consent. Participants can exit at any time during the questionnaire filling process.

### 2.4 Data collection and analysis

In order to ensure that this work obtains comprehensive and detailed data, a diversified set of data collection tools is employed.

**Figure 3:**
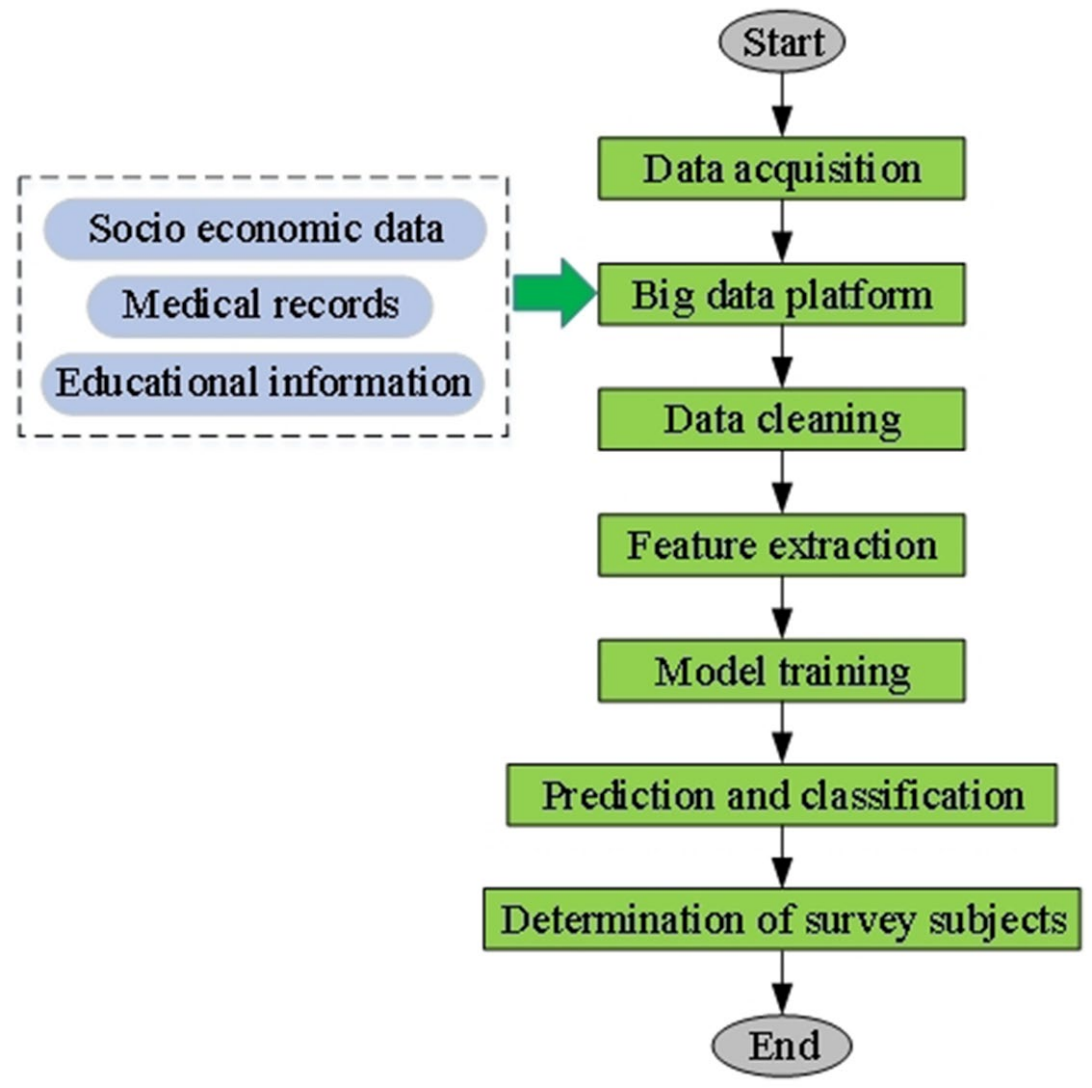
Flowchart of intelligent identification and classification process for the target population.

First, extensive use of big data analysis and artificial intelligence technology. As one of the artificial intelligence technologies, deep learning, combined with big data analysis techniques, can accurately identify low-income rural populations when designing intelligent recognition and classification models for target population identification. By aggregating comprehensive and diverse datasets through big data analysis, the model can extract features relevant to low-income rural populations with high precision. The application of deep learning algorithms further enhances this capability, allowing the model to learn and adapt to the specific characteristics of the target population through training. Intelligent algorithms perform feature extraction, data cleaning, and noise reduction, ensuring the quality of data input into the machine learning model. The trained model can quickly and accurately determine whether individuals belong to the low-income rural group through prediction and classification. Figure 3 illustrates the process of target population identification and classification.

Figure 3 illustrates the use of big data analysis (26), and deep learning (27), algorithms for identifying the target population. First, through big data analysis, this work aggregates multidimensional information, including socioeconomic, medical, and educational data, from various sources to form a large and diverse dataset. Intelligent algorithms perform feature extraction on this basis, identifying potential features related to rural low-income individuals and ensuring data quality through cleaning and noise reduction. Next, artificial intelligence technology (28, 29) is employed to establish a machine learning model. Training enables the model to learn and adapt to the characteristics of the rural low-income population. Ultimately, through prediction and classification, the model can rapidly and accurately determine whether individuals belong to the target group, ensuring the representativeness of the research sample and providing a reliable data foundation for this work.

Next, implementing a survey questionnaire is another crucial step for data collection. The questionnaire survey provides detailed data on participants’ basic information, medical conditions, educational background, and other aspects. The questionnaire content is designed based on the research objectives, focusing on the correlation between healthcare and future education, and covering multiple dimensions to ensure a comprehensive understanding of the situation of rural low-income populations.

### 2.5 Statistical analysis of data

In order to analyze the proposed target population recognition mechanism, it is compared with other neural network algorithms, including convolutional neural network (30) and gradient boosting decision tree (GBDT) (31). The accuracy and *F*-score (*F*_1_) values are used for population recognition accuracy evaluation. Equations 1, 2 present the calculation.

(1) $$Acc=\frac{\sum_{i=1}^{l}\frac{{TP}_{i}+{TN}_{i}}{{TP}_{i}+{FP}_{i}+{TN}_{i}+{FN}_{i}}}{l}$$

(2) $$F_{1}=\frac{2\mathrm{Precision}\times \mathrm{Recall}}{\mathrm{Precision}+\mathrm{Recall}}$$

Where TP is the number of true positive samples predicted as positive; FP is the number of false positive samples predicted as positive; FN is the number of false negative samples predicted as negative; TN is the number of true negative samples predicted as negative. Accuracy (ACC) is employed to measure the overall classification accuracy, and the *F*-measure is the weighted harmonic mean of precision and recall. Recall (Rec) in Equation 2 measures the coverage of positive samples, that is, the proportion of correctly classified positive samples to the total number of positive samples; precision (Pre) refers to the proportion of examples classified as positive that are actually positive. Equations 3, 4 provide the specific equations:

(3) $$\mathrm{Precision}=\frac{\sum_{i=1}^{l}\frac{{TP}_{i}}{{TP}_{i}+{FP}_{i}}}{l}$$

(4) $$\mathrm{Recall}=\frac{\sum_{i=1}^{l}\frac{{TP}_{i}}{{TP}_{i}+{FN}_{i}}}{l}$$

When analyzing the questionnaire survey results, the questionnaires are distributed offline, reaching a total distribution of 487. Four hundred fifty-nine completed questionnaires are received, achieving a high response rate of 94.25%. This indicates the effectiveness of the survey. To further ensure the validity of the questionnaire survey, during the statistical analysis of the questionnaires, the 459 valid questionnaires are carefully reviewed. Any questionnaires with significant issues are excluded. Ultimately, 436 valid questionnaires are obtained, resulting in a questionnaire validity rate of 94.99%. Statistical analysis is conducted using Stata 16 software.

## 3 Results and discussion

### 3.1 Accuracy analysis of population identification under different algorithms

**Figure 4:**
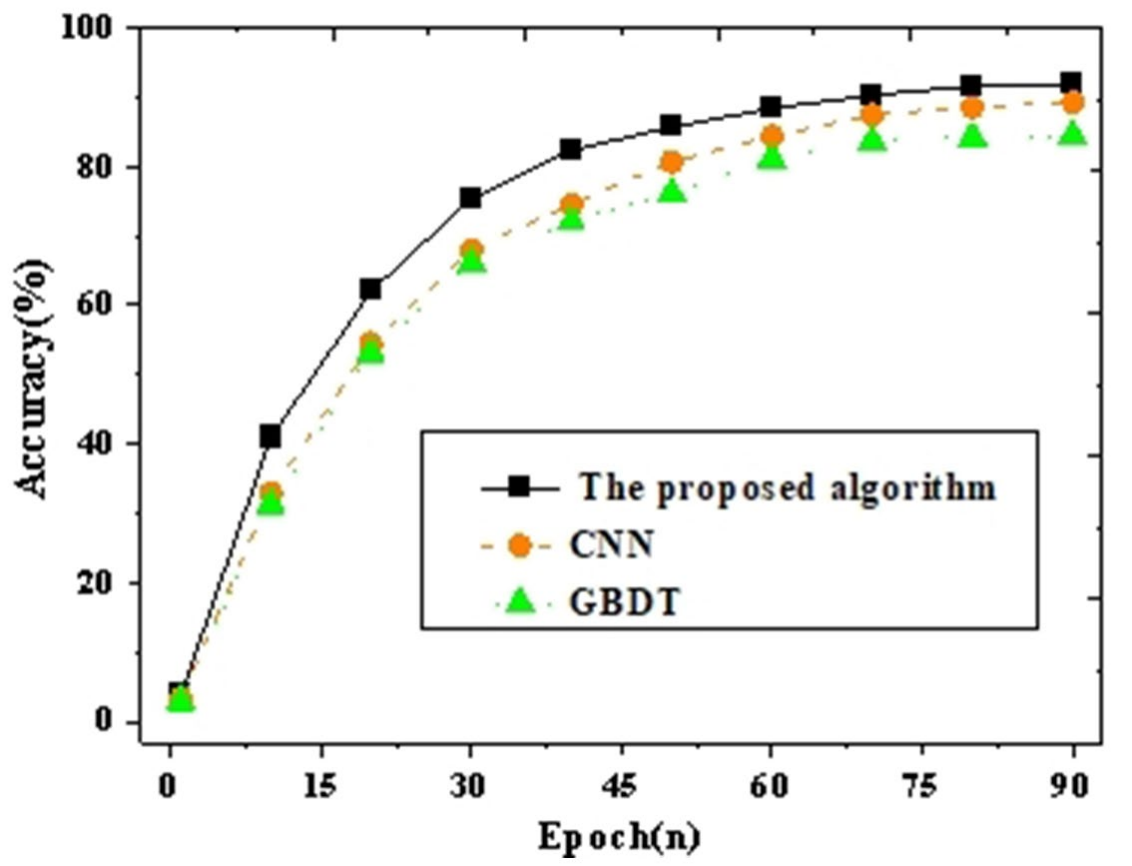
Accuracy curve graph compared with other neural networks.

**Figure 5:**
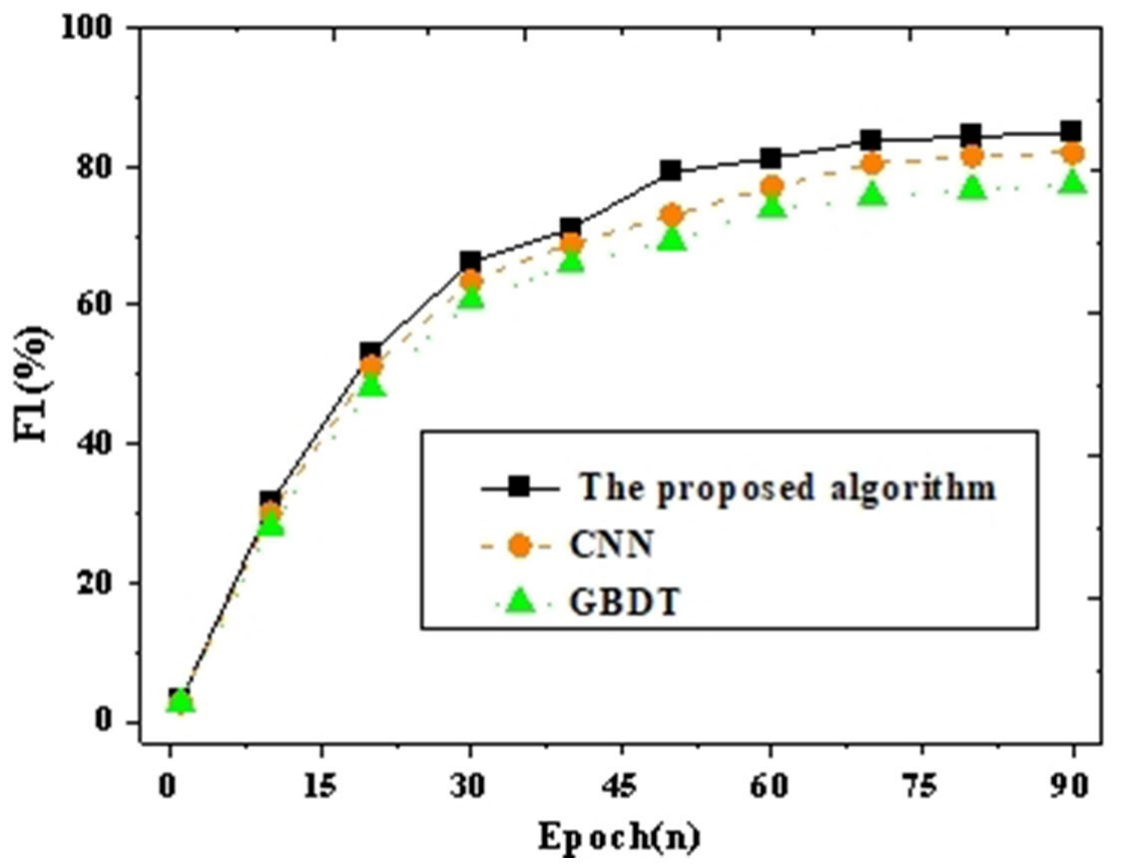
*F*_1_ curve graph compared with other neural networks.

The intelligent identification classification model for the target population is analyzed for accuracy and F_1_ value in comparison with CNN and GBDT. Figures 4, 5 present the results.

The results of accuracy and *F*_1_ values validate the comparison of the proposed target population identification mechanism with CNN and GBDT. Figures 4, 5 depict the outcomes. It can be observed that with an increase in iteration cycles, the trend of accuracy and *F*_1_ values initially experiences rapid growth, which stabilizes after reaching a certain value. Further comparative analysis reveals that the proposed method achieves an accuracy of 91.93% and an *F*_1_ value of 84.94% after 90 iterations. This is at least 2.65% higher in accuracy compared to other baseline neural network algorithms (such as CNN and GBDT). To verify whether these differences are statistically significant, a *t*-test is further conducted. The proposed model’s accuracy and *F*_1_ scores are compared pairwise with those of other algorithms, as shown in Table 2.

**Table 2:** Results of the *t*-test.

Comparison algorithm	*t* statistic	Degrees of freedom	*p*-value
Proposed model vs. CNN	3.21	198	<0.01
Proposed model vs. GBDT	2.98	198	<0.01

Table 2 illustrates whether the differences in accuracy and *F*_1_ scores between the proposed model and the CNN and GBDT algorithms are statistically significant. The *t* statistic indicates the magnitude of the difference, while the degrees of freedom reflect the sample size used to calculate the *t* statistic, minus one. The *p*-value represents the probability of observing the current or more extreme differences under the assumption of equal distributions. Generally, if the *p*-value is less than 0.05, the difference is considered statistically significant. Table 2 suggests that the differences in accuracy and *F*_1_ scores between the proposed model and both CNN and GBDT are statistically significant (*p*-values are both less than 0.01). This further validates the superiority of the proposed model. Therefore, it is evident that the proposed target population identification mechanism exhibits superior precision in identifying low-income populations compared to other neural network algorithms, demonstrating significantly enhanced performance.

### 3.2 Analysis of survey statistical results

**Figure 6:**
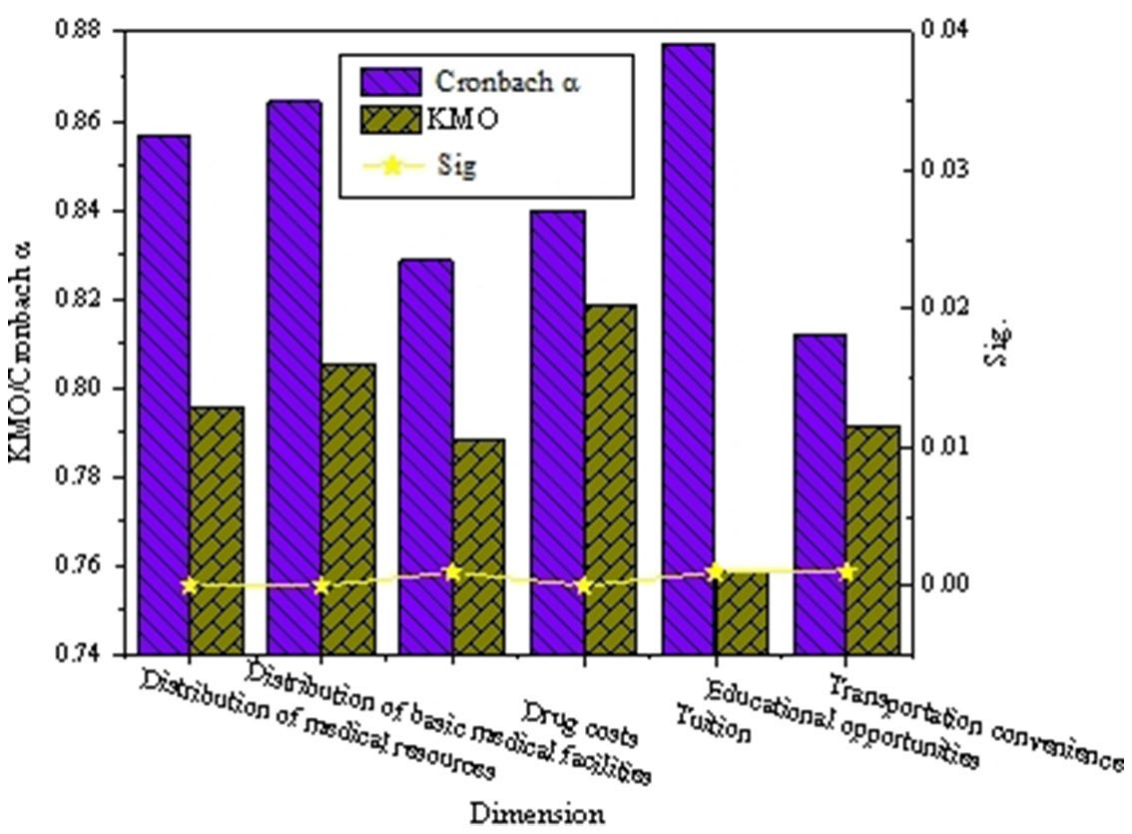
The diagram of the results of the reliability and validity test for the survey questionnaire.

A reliability and validity test is conducted on the survey results, and Figure 6 shows the results.

Figure 6 suggests that the Cronbach’s alpha values for each variable in the questionnaire are all greater than 0.800, KMO values are all greater than 0.259, and the significance (Sig.) values are all less than 0.050. This indicates that the survey questionnaire designed has significant internal consistency and stability, demonstrating high reliability. Therefore, the questionnaire used is considered rational and effective, forming a solid basis for further research.

**Figure 7:**
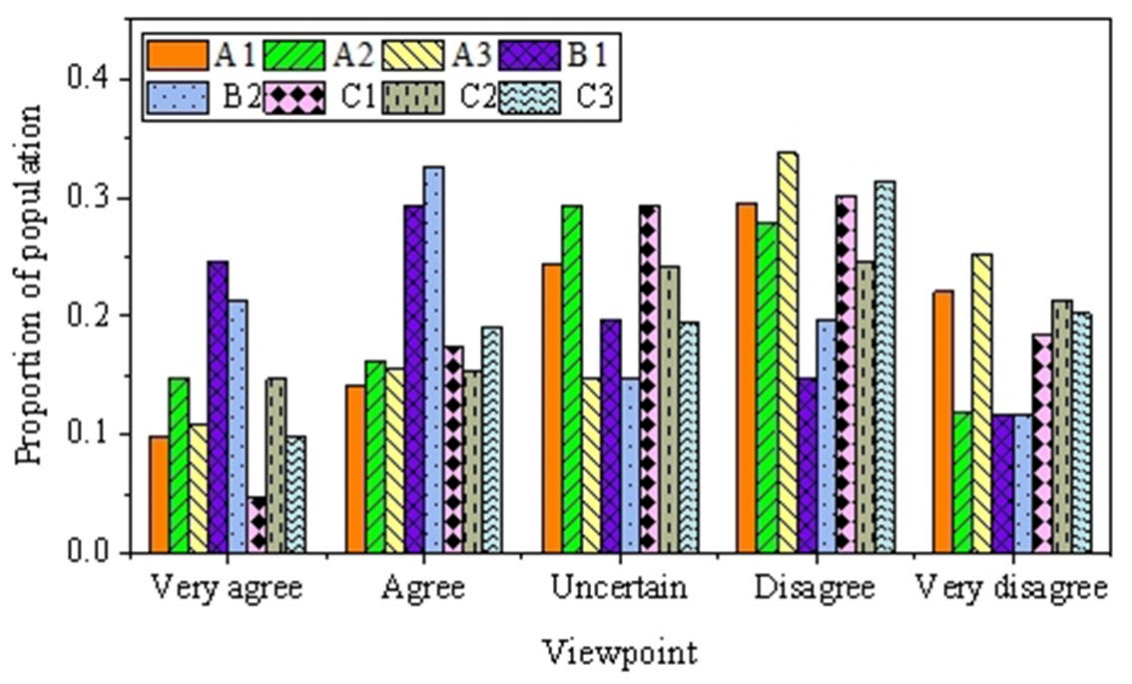
The survey results related to healthcare.

**Figure 8:**
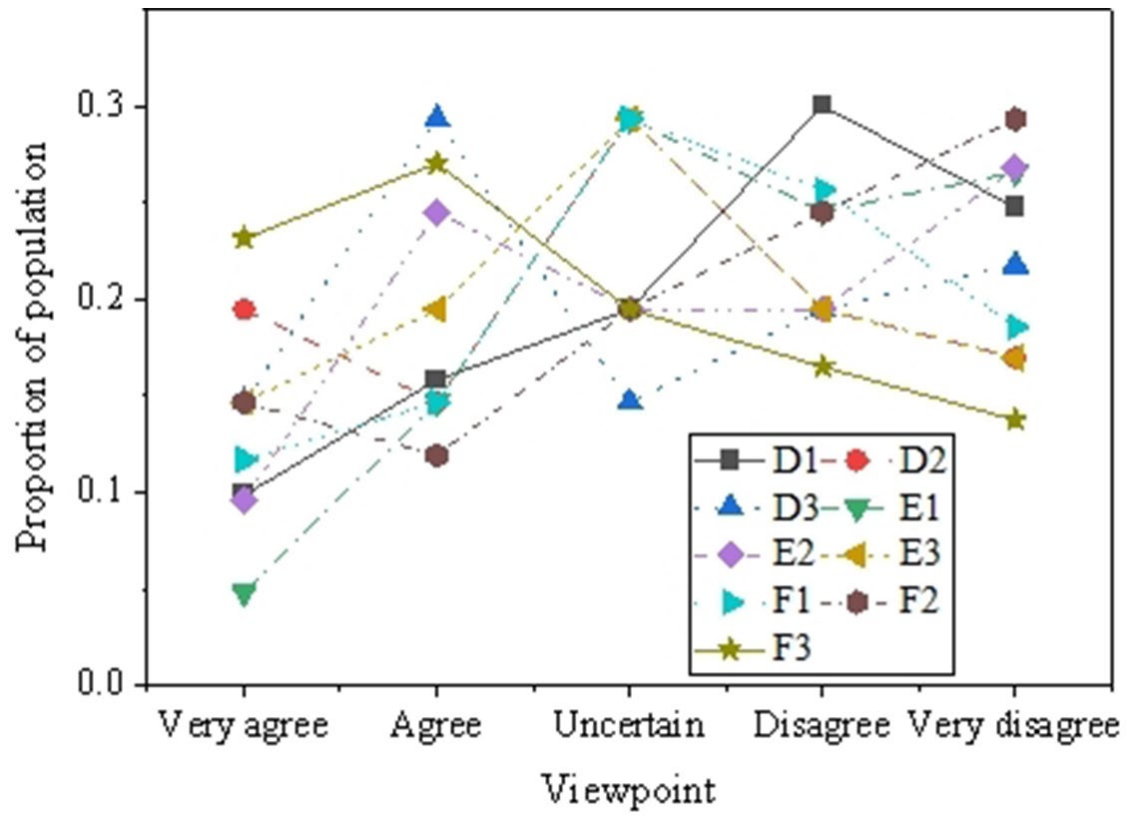
The survey results related to education.

Descriptive statistical analysis is conducted on the questionnaire results related to healthcare (distribution of medical resources, medication costs, distribution of basic medical facilities) and future education (tuition burden, differences in educational opportunities, transportation conditions). Figures 7, 8 present the results.

Figure 7 suggests that respondents opinions are relatively dispersed concerning the quality of service in nearby medical institutions, the completeness of service facilities, and the perceived distance to medical facilities. Regarding service quality, 29.59% of respondents express dissatisfaction with the quality of service in medical institutions. Similarly, 27.75 and 33.72% of respondents, respectively, indicate disagreement about the completeness of service facilities and the distance to medical facilities. Regarding the economic burden of treating chronic diseases and the cost of medication, a considerable proportion of respondents agree or strongly agree, indicating that some individuals find the economic burden of treating chronic diseases relatively manageable. However, a significant proportion of respondents express dissatisfaction concerning the existence of community health stations or clinics, the timeliness of service, and satisfaction with service quality. Specifically, 32.90% of respondents strongly disagree with the existence of community health services. Overall, there are certain issues in the field of healthcare, and respondents show relatively low satisfaction with some medical services.

Figure 8 displays the survey results reflecting respondents’ perspectives and attitudes towards education, revealing a range of viewpoints related to family economic conditions, the ability to pay for children’s education, school quality in the area, fairness in educational opportunities, and transportation for children’s schooling. In terms of family finances, a significant proportion of respondents express concerns about their ability to pay for their children’s education, with 30.05% considering their monthly income insufficient for tuition fees. Additionally, there is divergence among respondents regarding the perception of relatively lower tuition fees and the financial burden of tuition fees on the family economy, with 19.50 and 26.09% of respondents, respectively expressing strong disagreement. Regarding educational opportunities, the survey results indicate that 32.90% of respondents believe that schools in their area lack equivalent teaching staff, and 24.77% believe that school facilities in their area are inadequate. Furthermore, 43.67% of respondents agree that educational opportunities primarily depend on family economic conditions, indicating concerns about educational opportunities. A certain proportion of respondents consider transportation for children’s schooling convenient. However, there is disagreement regarding the impact of transportation on academic performance and family life, with 43.27 and 32.11% of respondents, respectively expressing agreement or strong agreement. The diversity of these viewpoints highlights the concerns of low-income rural populations regarding future education issues.

### 3.3 The discussion and analysis of the correlation between healthcare and education development

**Figure 9:**
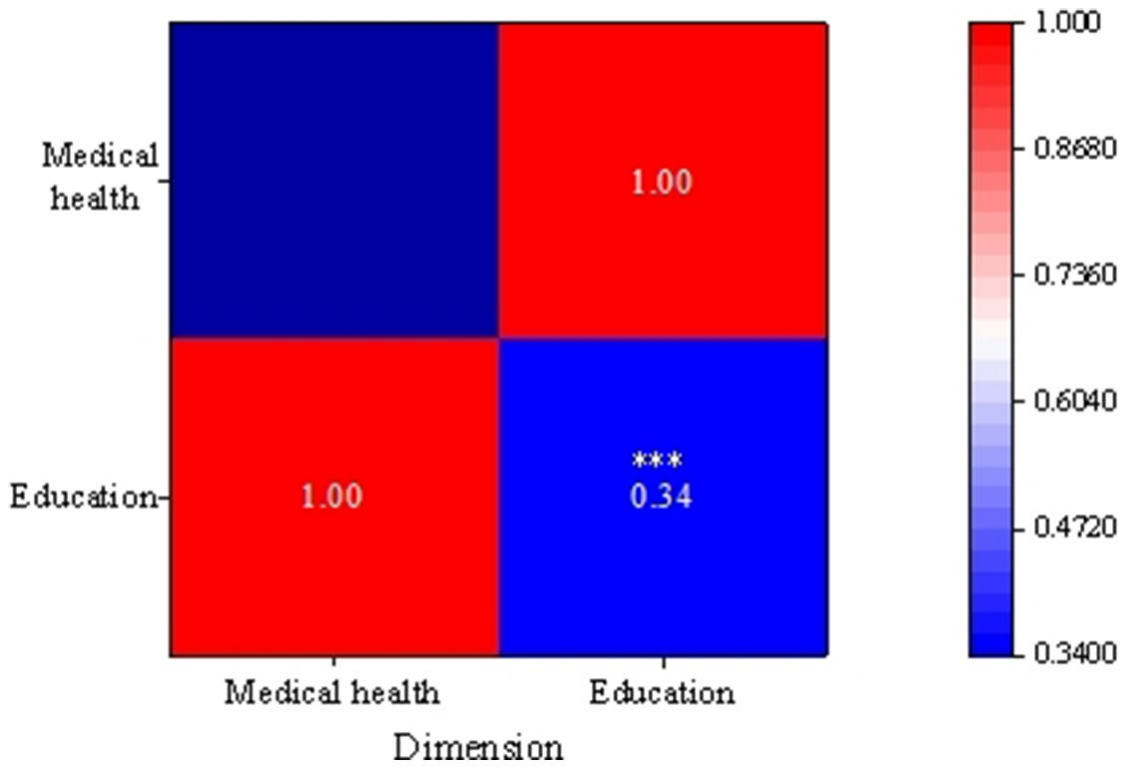
The correlation results between healthcare and education development.

Through the analysis of the questionnaire results on healthcare and future education, this work can delve into the correlation between healthcare and educational development. In terms of healthcare, the survey indicates that rural low-income populations express certain dissatisfaction with the quality of service, completeness of facilities, and distance to nearby medical institutions. Particularly, a significant proportion of respondents express dissatisfaction with community health services’ existence, timeliness, and quality. This may suggest that factors such as inadequate healthcare resources, uneven service levels, and distance limitations could impact the healthcare experience of rural low-income populations. Meanwhile, regarding future education, respondents present diverse perspectives on issues related to family finances, the ability to pay children’s school fees, the quality of schools in their area, fairness in educational opportunities, and the transportation of children to school. Figure 9 provides specific results of the correlation analysis.

Figure 9 reveals the importance of healthcare and future education among rural low-income individuals, indicating a positive correlation between them (*r* = 0.34). This suggests that in the process of normalizing assistance for rural low-income populations, there is a correlation and mutual influence between healthcare and future education. For instance, health issues may impact the economic status of low-income rural families, affecting their ability to pay for their children’s education. Simultaneously, the unfairness in educational opportunities may pose challenges for some families in accessing better healthcare services. Therefore, the complex relationship between health and education development can be better explored in future research to formulate more targeted policies and improvement measures, fostering the comprehensive development of rural low-income populations.

## 4 Discussion

Through analysis of the results, this work finds that the use of deep learning and big data analysis techniques for population identification achieves an accuracy of 91.93%, significantly outperforming the baseline algorithm of CNN. This finding aligns with the perspectives of Goel et al. (32) and Choi and Lim (33). Therefore, understanding the accuracy of the target population identification mechanism and the errors it generates is crucial for optimizing model performance and enhancing the precision of policy formulation. First, a highly accurate identification mechanism ensures that resources are more effectively allocated to rural low-income populations who truly need them, thereby improving the efficiency and effectiveness of aid programs. Then, by analyzing the sources of error, it is possible to identify and correct potential biases in the model, such as data collection bias, sample selection bias, or limitations inherent to the algorithm itself. This in turn enhances the fairness and reliability of the model. Additionally, the identification of errors can provide policymakers with insights into the current distribution of healthcare and educational resources, helping them to identify gaps and deficiencies in service provision, thus allowing for more targeted interventions. Finally, accurately identifying target populations is essential for assessing the impact of policies and evaluating their effectiveness. It provides a scientific basis for future policy adjustments and resource reallocation. This can ensure that policy formulation is grounded in actual data and objective analysis, thereby better serving rural low-income groups and promoting social equity and sustainable development. Therefore, a thorough understanding of the accuracy and errors in the target population recognition mechanism has significant benefits for enhancing the precision of aid programs, optimizing resource allocation, formulating effective policies, and promoting social justice.

Meanwhile, this work has explored the correlation between rural low-income populations in healthcare and future education, providing in-depth insights into the current situation of this specific demographic. Through the intelligent identification model developed using big data and deep learning algorithms, the work accurately identifies the target group. Moreover, it collects first-hand data through questionnaires on aspects such as healthcare resources, medication costs, satisfaction with basic healthcare facilities, educational burdens, disparities in educational opportunities, and transportation conditions. The analysis reveals that rural low-income populations exhibit low satisfaction with healthcare and have significant concerns regarding future education, primarily centered on economic burdens and inequalities in resource access. The work also uncovers a positive correlation between healthcare and education, indicating an interaction between health status and educational opportunities, which provides a basis for more targeted policy formulation. Furthermore, the intelligent identification model proposed outperforms existing algorithms in accuracy, offering a more precise tool for resource allocation and policy formulation. Thus, this work not only enhances the understanding of the needs of rural low-income populations but also provides valuable reference points for future research and policy formulation. It contributes to promoting equal opportunities in healthcare and education for these groups and fostering social equity and sustainable development.

Additionally, the research by Kadaei et al. (34) complements this work, as they proposed a new method to identify reverse logistics issues in smart buildings, with a particular focus on sustainable architecture. By integrating Kadaei’s et al. (34) approach, a more comprehensive understanding and optimization of resource allocation for rural low-income populations in healthcare and education can be achieved while considering the sustainability of the construction industry. This interdisciplinary approach not only enhances awareness of the needs of rural low-income populations but also provides new perspectives for the future development of smart buildings and sustainable environments.

Finally, to address the challenges faced by rural low-income populations in the education sector, it is recommended that relevant authorities implement the following measures to strengthen the effectiveness of educational poverty alleviation policies. First, it is essential to increase educational investment in rural areas to improve basic education facilities, ensuring that schools have qualified teachers and essential teaching resources; second, tuition reduction and educational subsidy policies should be implemented to alleviate the financial burden on low-income families, enabling their children to complete their studies successfully; additionally, distance education and technology-assisted teaching should be promoted to provide rural students access to a broader range of knowledge and information resources; simultaneously, vocational education and skills training need to be strengthened to enhance the employability of rural youth and provide them with more career development opportunities; finally, it is essential to establish and improve student funding systems to ensure that impoverished students do not lose educational opportunities due to financial difficulties. Through these specific and targeted measures, the ability of rural low-income populations to access educational resources can be effectively enhanced, promoting educational equity and yielding long-term benefits for them and society as a whole.

## 5 Conclusion

This work delves into the correlation between healthcare and future education among rural low-income populations by employing advanced population identification mechanisms and diverse data collection tools. Through survey questionnaires and data analysis, it systematically reveals the problems and challenges faced by this group in terms of healthcare and education. It also introduces a targeted population intelligent recognition classification model and demonstrates its significant advantages in low-income population identification classification accuracy by comparing it with other baseline neural network algorithms. However, this work has some limitations, such as regional scope constraints and the locality of survey questionnaire design. Despite obtaining meaningful results, practical applications may face challenges related to technology and privacy. Future research can expand and refine this work by broadening the research scope, deepening correlation analysis, and continuously improving technology and privacy protection. In conclusion, this work provides profound insights into the current situation of rural low-income populations and serves as a valuable reference foundation for future research and policy formulation.

## Data Availability

The original contributions presented in the study are included in the article/supplementary material, further inquiries can be directed to the corresponding author.
